# Remission and long-term remission of pediatric-onset systemic lupus erythematosus

**DOI:** 10.3389/fped.2023.1272065

**Published:** 2023-10-27

**Authors:** Yi-Chieh Chen, Chiann-Yi Hsu, Ming-Chin Tsai, Lin-Shien Fu, Yung-Chieh Huang

**Affiliations:** ^1^ Department of Pediatrics, Taichung Veterans General Hospital, Taichung, Taiwan; ^2^ Biostatistics Task Force of Taichung Veterans General Hospital, Taichung, Taiwan; ^3^ Department of Pediatrics, National Yang Ming Chiao Tung University, Taipei, Taiwan; ^4^ Department of Post-Baccalaureate Medicine, College of Medicine, National Chung Hsing University, Taichung, Taiwan; ^5^ Program in Translational Medicine, National Chung Hsing University, Taichung, Taiwan

**Keywords:** pediatrics, prognosis, remission, systemic lupus erythematosus, glucocorticoid

## Abstract

**Objectives:**

Systemic lupus erythematosus (SLE) is a chronic autoimmune disease with diverse clinical presentations and prognoses. Remission can be achieved with or without glucocorticoid (GC) use, and several recent studies have suggested that long-term remission can be achieved in a small portion of patients. Nevertheless, few studies have investigated remission or long-term remission in the pediatric-onset SLE subgroup. This study analyzed the characteristics and factors associated with long-term remission and GC use in pediatric-onset SLE.

**Methods:**

We enrolled 226 patients aged <18 years who received a diagnosis of SLE between January 2006 and December 2016. Three remission condition groups were defined: (A) complete remission, (B) clinical remission off GCs, and (C) clinical remission on GCs. Long-term remission was defined as remission for more than 5 years. We analyzed the treatment durations before remission, durations of remission, and risk factors for non-remission with persistent GC use.

**Results:**

During follow-up, 8 patients (3.5%) achieved complete remission, 35 patients (15.5%) achieved clinical remission off GCs, and 93 patients (41.2%) achieved clinical remission on GCs. In groups A, B, and C, 12.5%, 68.6%, and 65.6% of patients, respectively, remained in remission for >1 year.

**Conclusion:**

This study assessed remission of pediatric-onset SLE. Up to 60.2% of patients had clinical remission after treatment, and 19% of patients achieved remission off GCs. Long-term remission is rarer in pediatric-onset SLE than in adult-onset SLE.

## 1. Introduction

Systemic lupus erythematosus (SLE) is a chronic autoimmune disease. Although the prognosis and survival rate of patients with SLE have improved significantly, relapses and are still common (1). Approximately 10%–20% of patients with SLE have pediatric-onset SLE (2, 3). Pediatric-onset SLE is more active, more frequently involves the kidneys and neuropsychiatry, and causes damage earlier than adult-onset SLE. Immunosuppressive treatment is more common among patients with pediatric-onset SLE than among those with adult-onset SLE (2, 4–7). The prognoses, including long-term survival and renal outcomes, are worse among patients with pediatric-onset SLE than among those with adult-onset SLE (8–10).

Achieving remission is possible for patients with SLE. Studies have reported long-term remission in patients with SLE; however, these studies have provided varied definitions of “long-term” or “prolonged” (1, 11–14). Only a small portion of patients with adult-onset SLE achieve long-term remission. Remission is even rarer among patients with pediatric-onset SLE than among those with adult-onset SLE. Achieving remission is one of the primary goals of SLE treatment. Remission can influence health-related quality of life and may protect patients against some of the subsequent morbidities of SLE, including flare-up events and cardiovascular and renal diseases (15–17). Though there were several works of literatures reporting the remission of SLE adult patients, few reported the rates or characteristics of remission for pediatric-onset SLE.

This study aimed to clarify the characteristics of remission for pediatric-onset SLE. We followed the definition of SLE remission from the literature of Zen et al. (13), which considers clinical conditions, serological activities, and treatments. We analyzed the remission of pediatric-onset SLE, including its rate, duration, and predictors.

## 2. Materials and methods

### 2.1. Patients

We used data from the Taichung Veterans General Hospital Research Database, which is managed by the Clinical Informatics Research & Development Center of Taichung Veterans General Hospital. Patient information was anonymized before being made available for our research. The research protocol was approved by the Institutional Review Board of Taichung Veterans General Hospital (IRB number: CE20204A).

Patients who were admitted to our wards or visited our outpatient clinic and who received a diagnosis of SLE between January 2006 and December 2016 were included. Patients who fulfilled the 1997 ACR (18) or 2012 SLICC (19) classification criteria were defined as having SLE. Patients aged >18 years were excluded. Laboratory data were retrospectively analyzed. Treatments included glucocorticoid (GC), high-dose GC, anti-malarial, immunosuppressive, and biological drug therapies.

A total of 329 patients were initially screened. We excluded patients with less than three visits to the outpatient clinic, those with follow-up periods <6 months, those who did not receive a GC prescription, and those who were lost to follow-up in our hospital. A total of 226 patients were subsequently included in the analysis. We collected their serological and laboratory data (including complete blood count, differential count, urine analysis, and biochemistry function) and their data on clinical disease activity and complications.

### 2.2. Disease activity and definitions of remission

Three definitions of remission were considered according to the disease activity and treatment: (A) complete remission, (B) clinical remission off GCs, and (C) clinical remission on GCs (Table 1). GCs are the mainstay of treatment in pediatric patients with SLE (8).

(A) Complete remission: no clinical or serological disease activity; GC-free and immunosuppressant-free; and antimalarials were allowed.
(B) Clinical remission off GC: serologically active, clinically quiescent disease; GC-free and immunosuppressant-free; and antimalarials were allowed.
(C) Clinical remission on GC: serologically active, clinically quiescent disease; immunosuppressant-free; GCs and antimalarials were allowed; and long-term remission was defined as remission for more than 5 years.

**Table 1:** Definitions of remission according to clinical, serological and therapeutic status.

	Disease activity		Treatment		
	Clinical	Serology	Antimalarials	Steroid	Immunosuppressants
Complete remission	—	—	±	—	—
Clinical remission off steroids	—	±	±	—	—
Clinical remission on steroids	—	±	±	+	—

### 2.3. Clinical conditions and laboratory data

Clinical conditions were judged based on laboratory data and diagnoses in inpatient and outpatient medical records. Laboratory data included complete blood count, renal function, liver function, C-reactive protein, erythrocyte sedimentation rate, and urine analysis results. Serological data included the titers of antinuclear antibodies and anti–double stranded DNA (anti-dsDNA) antibodies; Anti-dsDNA would be considered positive if it is positive in anytime during the disease course. The highest and lowest C3 and C4 indicated the highest and lowest level in anytime during the disease course. Treatments included nonsteroidal anti-inflammatory drugs, GCs, disease-modifying antirheumatic drugs, and immunosuppressives and biological therapies. Antimalarials were allowed in all remission groups.

### 2.4. Statistical analysis

Analyses were performed using Statistical Package for the Social Sciences (IBM SPSS version 22.0; New York, USA). Normal distribution of the data was verified with the Kolmogorov–Smirnov test. Continuous data are expressed as median and interquartile range. Continuous data were compared using the Kruskal–Wallis test and Mann–Whitney U test. Categorical data are expressed as numbers and percentages. Categorical data were compared using the chi-square and Fisher's exact tests. Statistical significance was indicated by *p* values of <0.05. Cox regression analysis was performed to identify baseline patient characteristics (including laboratory data and clinical conditions) associated with the time to remission, with adjustment for baseline disease activity and treatment. We examined one variable at a time with Cox regression, including demographic and immunological characteristics and specific types of disease activity. Patient characteristics, especially the initial and lowest C3/C4 levels, were analyzed, and the results are expressed as hazard ratios and 95% conﬁdence intervals.

## 3. Results

### 3.1. Patient characteristics

Among the 226 patients, 8 (3.5%) achieved complete remission, 35 (15.5%) achieved clinical remission off GCs, and 93 (41.2%) achieved clinical remission on GCs. In total, 90 patients (39.8%) did not achieve any level of remission (Table 2). Characteristics of these four groups are summarized in Table 2, showing the mean age at the time of diagnosis, gender distribution, serological data (antinuclear antibodies and anti-dsDNA antibodies), and clinical presentations. The mean age at initial diagnosis was 13.9 ± 3.1 years. The male-to-female ratio was 1:7.4. The three clinical remission groups exhibited differences in the lowest antinuclear antibodies titers (*p* = 0.008), the highest C3 (*p* < 0.001) and highest C4 (*p* = 0.001) levels, the percentages of ever positive anti-dsDNA antibodies (*p* = 0.038), leukopenia (*p* = 0.002), hematuria (*p* = 0.034), and seizure (*p* = 0.002).

**Table 2:** Patient demographics and clinical characteristics overall and according to different types of remission.

	No-remission (*n* = 90)		Complete remission (*n* = 8)		Clinical remission off steroids (*n* = 35)		Clinical remission on steroids (*n* = 93)		Overall (*n* = 226)		*p* value
Age of SLE diagnosis	14.0	±3.1	13.5	±2.5	14.3	±2.7	13.6	±3.3	13.9	±3.1	0.605
Gender (male:female)	9: 81		0: 8		5: 30		13: 80		27: 199		0.363
Initial ANA titer											0.151
1:40	0	(0.0%)	0	(0.0%)	1	(2.9%)	3	(3.2%)	4	(1.8%)	
1:80	5	(5.6%)	3	(37.5%)	1	(2.9%)	9	(9.7%)	18	(8.0%)	
1:160	17	(18.9%)	3	(37.5%)	3	(8.6%)	16	(17.2%)	39	(17.3%)	
1:320	18	(20.0%)	1	(12.5%)	6	(17.1%)	15	(16.1%)	40	(17.7%)	
1:640	13	(14.4%)	0	(0.0%)	9	(25.7%)	17	(18.3%)	39	(17.3%)	
1:1,280	21	(23.3%)	1	(12.5%)	7	(20.0%)	15	(16.1%)	44	(19.5%)	
1:2,560	16	(17.8%)	0	(0.0%)	8	(22.9%)	16	(17.2%)	40	(17.7%)	
1:5,120	0	(0.0%)	0	(0.0%)	0	(0.0%)	2	(2.2%)	2	(0.9%)	
Lowest ANA titer											0.008**
1:40	4	(4.4%)	3	(37.5%)	5	(14.3%)	12	(12.9%)	24	(10.6%)	
1:80	12	(13.3%)	5	(62.5%)	5	(14.3%)	18	(19.4%)	40	(17.7%)	
1:160	15	(16.7%)	0	(0.0%)	5	(14.3%)	16	(17.2%)	36	(15.9%)	
1:320	14	(15.6%)	0	(0.0%)	8	(22.9%)	16	(17.2%)	38	(16.8%)	
1:640	14	(15.6%)	0	(0.0%)	8	(22.9%)	11	(11.8%)	33	(14.6%)	
1:1,280	19	(21.1%)	0	(0.0%)	4	(11.4%)	9	(9.7%)	32	(14.2%)	
1:2,560	12	(13.3%)	0	(0.0%)	0	(0.0%)	10	(10.8%)	22	(9.7%)	
1:5,120	0	(0.0%)	0	(0.0%)	0	(0.0%)	1	(1.1%)	1	(0.4%)	
Highest ANA titer											0.210
1:80	1	(1.1%)	0	(0.0%)	1	(2.9%)	1	(1.1%)	3	(1.3%)	
1:160	13	(14.4%)	2	(25.0%)	2	(5.7%)	16	(17.2%)	33	(14.6%)	
1:320	24	(26.7%)	4	(50.0%)	4	(11.4%)	15	(16.1%)	47	(20.8%)	
1:640	11	(12.2%)	1	(12.5%)	6	(17.1%)	19	(20.4%)	37	(16.4%)	
1:1,280	23	(25.6%)	1	(12.5%)	10	(28.6%)	15	(16.1%)	49	(21.7%)	
1:2,560	18	(20.0%)	0	(0.0%)	11	(31.4%)	24	(25.8%)	53	(23.5%)	
1:5,120	0	(0.0%)	0	(0.0%)	1	(2.9%)	3	(3.2%)	4	(1.8%)	
Anti-dsDNA antibodies (positive %)	57	(63.3%)	1	(12.5%)	18	(51.4%)	54	(58.1%)	130	(57.5%)	0.038*
Initial C3 (mg/dl)	56.1	±43.7	62.3	±55.7	58.8	±52.6	52.3	±47.4	55.2	±47.0	0.662
Lowest C3 (mg/dl)	46.4	±40.6	39.3	±41.0	37.1	±36.7	45.6	±42.0	44.3	±40.5	0.902
Highest C3 (mg/dl)	97.3	±34.8	112.8	±15.4	125.5	±25.9	111.1	±37.8	108.2	±35.6	<0.001**
Initial C4 (mg/dl)	8.5	±8.7	12.8	±11.2	9.5	±10.8	9.6	±9.3	9.3	±9.4	0.793
Lowest C4 (mg/dl)	6.5	±8.2	8.9	±8.9	5.5	±7.7	6.7	±7.9	6.5	±8.0	0.492
Highest C4 (mg/dl)	20.5	±12.0	24.0	±9.3	30.6	±13.0	24.8	±11.2	24.0	±12.2	0.001**
Leukopenia [number(%)]	44	(48.9%)	1	(12.5%)	27	(77.1%)	56	(60.2%)	128	(56.6%)	0.002**
Thrombocytopenia [number(%)]	38	(42.2%)	1	(12.5%)	21	(60.0%)	40	(43.0%)	100	(44.2%)	0.072
Hematuria [number(%)]	46	(51.1%)	6	(75.0%)	26	(74.3%)	63	(67.7%)	141	(62.4%)	0.034*
Pyuria [number(%)]	64	(71.1%)	7	(87.5%)	30	(85.7%)	69	(74.2%)	170	(75.2%)	0.310
Proteiuria [number(%)]	22	(24.4%)	2	(25.0%)	9	(25.7%)	23	(24.7%)	56	(24.8%)	0.999
CVA [number(%)]	0	(0.0%)	0	(0.0%)	2	(5.7%)	1	(1.1%)	3	(1.3%)	0.089
Headache [number(%)]	7	(7.8%)	1	(12.5%)	1	(2.9%)	12	(12.9%)	21	(9.3%)	0.321
Organic brain syndrome [number(%)]	0	(0.0%)	0	(0.0%)	0	(0.0%)	1	(1.1%)	1	(0.4%)	0.697
Psychosis [number(%)]	1	(1.1%)	0	(0.0%)	2	(5.7%)	0	(0.0%)	3	(1.3%)	0.088
Seizure [number(%)]	1	(1.1%)	2	(25.0%)	2	(5.7%)	2	(2.2%)	7	(3.1%)	0.002**
Visual disturbance [number(%)]	0	(0.0%)	0	(0.0%)	0	(0.0%)	1	(1.1%)	1	(0.4%)	0.697
Vasculitis [number(%)]	2	(2.2%)	0	(0.0%)	3	(8.6%)	5	(5.4%)	10	(4.4%)	0.388

Chi-Square test. Kruskal Wallis test.

Continuous data were expressed median and IQR.

Categorical data were expressed number and percentage.

* *p* < 0.05.

** *p* < 0.01.

### 3.2. Treatment duration before remission

In the complete remission group, the treatment duration before remission was 899 ± 668.8 days. In the clinical remission off GCs group, this duration was 840.9 ± 708.7 days. By contrast, 72 patients (77.4%) achieved clinical remission on GCs within 1 year, with a mean duration of treatment of 312 ± 609.7 days. The difference in the treatment duration before remission between the clinical remission on GCs group and the other groups was statistically significant (*p* < 0.001).

### 3.3. Duration of remission

**Figure 1:**
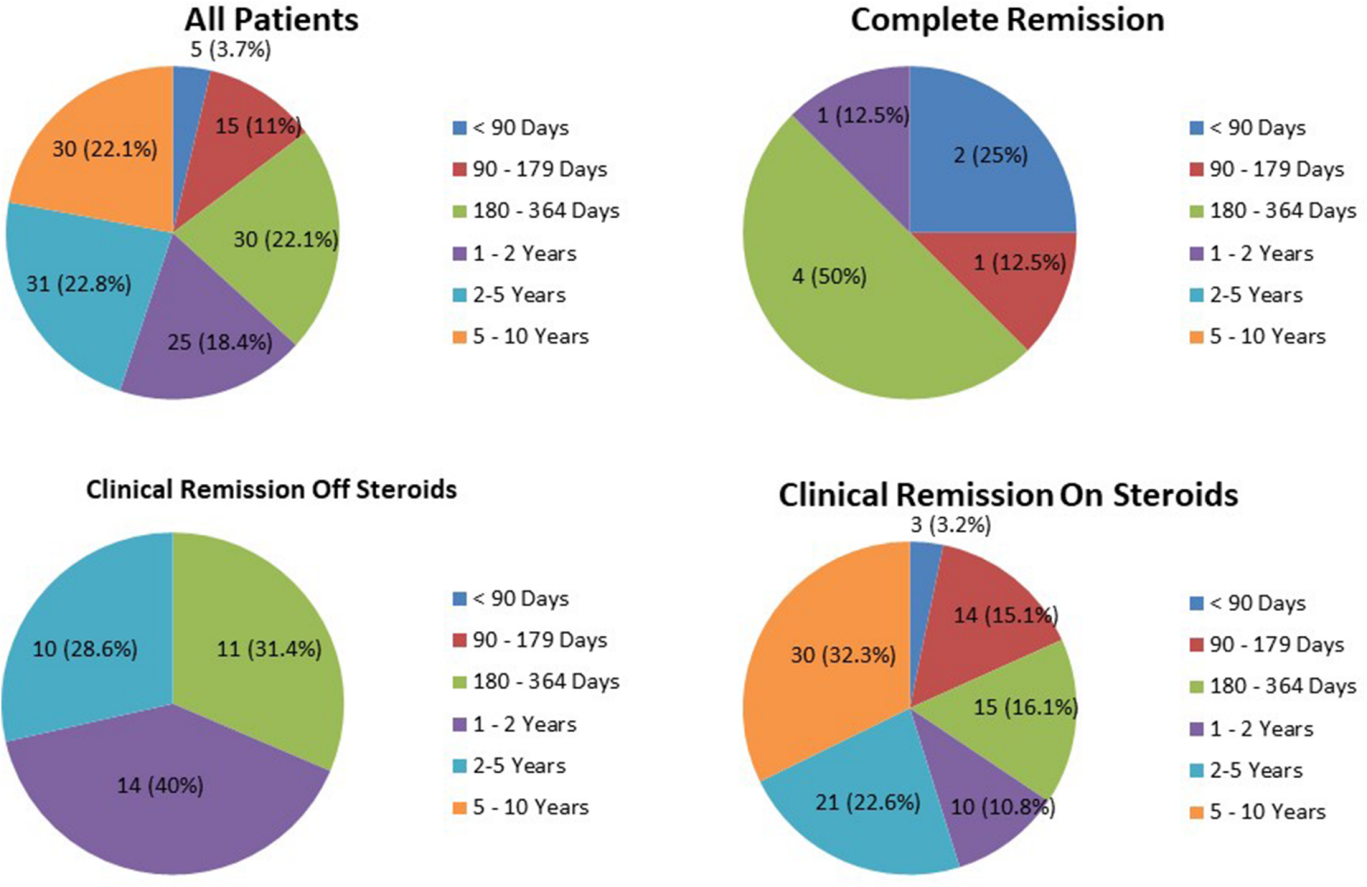
The duration of remission of the three remission groups.

The duration of remission of the three remission groups is provided in Figure 1. The average duration of complete remission was 248.1 ± 141.43 days. Only one patient (12.5%) remained in complete remission for more than 1 year (1.29 years). In the clinical remission off GCs group, 24 patients (68.6%) remained in remission for more than 1 year. In the clinical remission on GCs group, 61 patients (65.6%) remained in remission for periods exceeding 1 year; 30 patients (32.3%) remained in remission for more than 5 years (long-term remission).

### 3.4. Characteristics of patients who achieved complete remission

Compared with patients who did not achieve complete remission, the eight patients who achieved complete remission had lower initial antinuclear antibody titers (*p* = 0.043) and the lowest antinuclear antibody titers (*p* = 0.004). Only one patient (12.5%) who achieved complete remission had positive anti-dsDNA antibodies. By contrast, among the 218 patients who did not achieve complete remission, 129 (59.2%) had positive anti-dsDNA antibodies (*p* = 0.011). No significant differences were observed in C3 and C4 levels between these two groups. The complete remission group exhibited a lower rate of leukopenia (12.5% vs. 58.3%, *p* = 0.023) but a higher rate of seizure (25% vs. 2.3%, *p* = 0.021). Furthermore, no significant differences were observed in the rates of cardiovascular accident, headache, organic brain syndrome, psychosis, visual disturbance, and vasculitis.

### 3.5. Analyses of patients who achieved clinical remission

We further divided patients who achieved clinical remission into two subgroups, off GC and on GC, as mentioned earlier in this article. One subgroup discontinued GCs for a period, and the other subgroup used GCs throughout the study period. The characteristics of these two subgroups are shown in Table 3. No significant differences in patient characteristics, namely serological findings, complement C3/C4 levels, laboratory data, or severe clinical manifestations, were observed between these two subgroups. However, in Cox regression, we observed that when the initial C3, initial C4, and lowest C4 levels were increased by 1, the probability of discontinuing GCs was increased by 1.01-fold, 1.07-fold, and 1.07-fold respectively after age and gender adjustment (Table 4).

**Table 3:** Patient demographics and cumulative disease manifestations according to GC use.

	On steroid remission (*n* = 93)		Off steroid remission (*n* = 43)		*p* value
Age of SLE diagnosis	13.6 ± 3.3		14.1 ± 2.7		0.461
Gender (male: female)	13: 80		5: 38		0.917
Initial ANA titer					0.984
1:40	3	(3.2%)	1	(2.3%)	
1:80	9	(9.7%)	4	(9.3%)	
1:160	16	(17.2%)	6	(14.0%)	
1:320	15	(16.1%)	7	(16.3%)	
1:640	17	(18.3%)	9	(20.9%)	
1:1,280	15	(16.1%)	8	(18.6%)	
1:2,560	16	(17.2%)	8	(18.6%)	
1:5,120	2	(2.2%)	0	(0.0%)	
Lowest ANA titer					0.374
1:40	12	(12.9%)	8	(18.6%)	
1:80	18	(19.4%)	10	(23.3%)	
1:160	16	(17.2%)	5	(11.6%)	
1:320	16	(17.2%)	8	(18.6%)	
1:640	11	(11.8%)	8	(18.6%)	
1:1,280	9	(9.7%)	4	(9.3%)	
1:2,560	10	(10.8%)	0	(0.0%)	
1:5,120	1	(1.1%)	0	(0.0%)	
Highest ANA titer					0.759
1:80	1	(1.1%)	1	(2.3%)	
1:160	16	(17.2%)	4	(9.3%)	
1:320	15	(16.1%)	8	(18.6%)	
1:640	19	(20.4%)	7	(16.3%)	
1:1,280	15	(16.1%)	11	(25.6%)	
1:2,560	24	(25.8%)	11	(25.6%)	
1:5,120	3	(3.2%)	1	(2.3%)	
Anti-dsDNA antibodies	54	(58.1%)	19	(44.2%)	0.185
Initial C3 (*n* = 253)	52.3	±47.4	59.5	±52.5	0.243
Lowest C3 (*n* = 253)	45.6	±42.0	37.6	±37.0	0.827
Highest C3 (*n* = 253)	111.1	±37.8	123.0	±24.6	0.237
Initial C4 (*n* = 265)	9.6	±9.3	10.2	±10.8	0.852
Lowest C4 (*n* = 265)	6.7	±7.9	6.2	±8.0	0.513
Highest C4 (*n* = 265)	24.8	±11.2	29.3	±12.6	0.074
Leukopenia [number(%)]	56	(60.2%)	28	(65.1%)	0.721
Thrombocytopenia [number(%)]	40	(43.0%)	22	(51.2%)	0.482
Hematuria [number(%)]	63	(67.7%)	32	(74.4%)	0.557
Pyuria [number(%)]	69	(74.2%)	37	(86.0%)	0.184
Proteinuria [number(%)]	23	(24.7%)	11	(25.6%)	1.000
CVA [number(%)]	1	(1.1%)	2	(4.7%)	0.235
Headache [number(%)]	12	(12.9%)	2	(4.7%)	0.224
Organic brain syndrome [number(%)]	1	(1.1%)	0	(0.0%)	1.000
Psychosis [number(%)]	0	(0.0%)	2	(4.7%)	0.098
Seizure [number(%)]	2	(2.2%)	4	(9.3%)	0.079
Visual disturbance [number(%)]	1	(1.1%)	0	(0.0%)	1.000
Vasculitis [number(%)]	5	(5.4%)	3	(7.0%)	0.708

**Table 4:** Variables independently associated with remission off GCs based on Cox regression controlling for disease activity and treatments.

	Univariate			Adjusted for age and gender		
	HR	(95% CI)	*p* value	HR	95% CI	*p* value
Initial C3	1.01	(1.003–1.02)	0.003**	1.01	(1.004–1.02)	0.002**
Initial C4	1.06	(1.02–1.09)	0.001**	1.07	(1.03–1.11)	0.001**
Lowest C4	1.06	(1.02–1.11)	0.007**	1.07	(1.02–1.12)	0.004**

Cox regression.

** *p* < 0.01.

## 4. Discussion

Few studies have investigated remission among patients with pediatric-onset SLE. Studies have mainly focused on renal involvement (9, 10). In the present study, 60.2% of patients achieved some level of remission.

The remission rate varies among studies involving patients 18 years or older. In a review in 2019 (12), Ruiz-Irastorza et al. reported that the 1-year remission rate ranged from 9.2% to 54.6%, regardless of treatment. However, no strict or universal definitions of remission or long-term remission were applied; therefore, the results may not be comparable between different studies. The definition of the remission of SLE was revised in 2021 by an international task force known as the DORIS Task Force (16). The definition included (1) clinical SLE disease activity index = 0 (2), Physician Global Assessment score <0.5 (on a scale of 0–3) (3), prednisolone dose equal to or less than 5 mg/day, and (4) stable use of antimalarials, immunosuppressives, and biologics. Serology was not included in the definition. Whether this definition can be applied to pediatric-onset SLE requires further validation.

In the further analysis of the characteristics of patients who achieved complete remission, our study revealed that the complete remission group had lower initial antinuclear antibody titers, fewer positive anti-dsDNA antibodies, and a lower rate of leucopenia. Although statistically significant, our relatively small sample size may lead to different results compared with other studies. ANA titer does not reflect disease activity. However, in some studies, ANA titers were used to predict the highest cumulative disease activity and immunosuppressants/ biologics use (20).

Wilhelm et al. (14) observed that both baseline positive anti-dsDNA and baseline low C4 levels are negative predictors of complete remission with and without treatment. In our study, we found that in patients with pediatric-onset SLE, initial complement C3 and C4 levels were associated with the discontinuation of GCs, which is similar to the findings of Wilhelm et al. When the initial C3, initial C4, and lowest C4 levels were increased by 1, the probability of discontinuing GCs was increased by 1.01-fold, 1.07-fold, and 1.07-fold respectively after age and gender adjustment in this study.

Long-term remission is not common, even in adult-onset SLE. A recent review suggested that among patients with SLE, approximately 15% can achieve prolonged remission, 70% have a relapse-remitting disease course, and the remaining 15% have persistently active disease (21). Zen et al. (13) found that 37% of patients achieved prolonged (more than 5 years) remission, 7.1% achieved prolonged complete remission, 14.7% achieved prolonged clinical remission off GCs, and 15.6% achieved prolonged clinical remission on GCs. In an observational study including two cohorts with similar SLE manifestations at diagnosis (12), Ruiz-Irastorza et al. reported a comparison of remission rates between two different treatment strategies. Patients recruited in the longitudinal Cruces Lupus Cohort, with more frequently treated with hydroxychloroquine, methotrexate, and pulse methylprednisolone, were more likely to have prolonged (4 years) clinical remission than the Bordeaux Lupus Cohort (70% and 28% respectively). In a retrospective cohort study involving 2,121 patients that was conducted by Jakez-Ocampo et al. (11), only 44 patients achieved sustained remission for more than 10 years. Tselios et al. described an atypical monophasic course (22). They also found that 10.1% of patients with SLE achieved prolonged clinical remission for more than 10 years, and that 20 patients (7.5%) achieved remission for the entire follow-up, averaging 18 years.

Various studies have reported different findings for baseline (first visit) clinical or immunological variables between patients with SLE who did and did not achieve prolonged remission. Tselios et al. (22) reported no clinical or immunological differences between patients with SLE who did and did not achieve prolonged remission. In a cohort study, Jakez-Ocampo et al. (11) found that older age at disease onset (with a *p* value of 0.055) was associated with prolonged remission (more than 10 years). This finding may partially explain why prolonged remission was rare in our study, which focused on patients with pediatric-onset SLE.

Biological agents, such as rituximab and belimumab, are emerging as new treatment choices for SLE (21). Both rituximab and belimumab demonstrated favorable safety and efficacy for treating pediatric-onset SLE (23, 24). Other therapies, such as anti-interferon monoclonal antibodies and Janus kinase inhibitors, are being investigated for treating pediatric-onset SLE (25). Whether biologics can affect remission and long-term remission rates in both pediatric-onset and adult-onset SLE is unclear.

The present study has several limitations. This was a retrospective, single-center cohort study. The number of included patients was relatively small, leading to possible insufficient statistical power for some of our results. All of our patients were Asian/Taiwanese; thus, our findings may not be generalizable to other ethnic groups. The definition of remission used in our study may differ from that used in other studies; thus, the results may not be comparable with those of other studies; for example, in our study, immunosuppressants were not allowed for any degree of remission, whereas they were allowed in other studies and according to the 2021 definition established by the DORIS Task Force. The status of lupus nephritis and renal remission, occurring more often and more severely among patients with pediatric-onset SLE than among those with adult-onset SLE (9, 26), was not included in this study due to poor correlations observed between systemic and renal activities (9). We checked the patient's ANA titers every 6–12 months in follow-ups, but not strictly; this may affect our interpretation of the lowest ANA titer in the study.

In conclusion, our study showed that more than 60% of patients with pediatric-onset SLE achieved some degree of remission, but only a small portion achieved long-term remission. Further observational studies focusing on pediatric-onset SLE are necessary for a more accurate prediction of clinical outcomes in this patient group.

## Data Availability

The raw data supporting the conclusions of this article will be made available by the authors, without undue reservation.
